# Supplementary material for: Qualitative and Quantitative Detection of Multiple Sexually Transmitted Infection Pathogens Reveals Distinct Associations with Cervicitis and Vaginitis
Source: Microbiol Spectr. 2022 Oct 31;10(6):e01966-22. doi: 10.1128/spectrum.01966-22 (PMC9769840; doi:10.1128/spectrum.01966-22)
Supplement: Supplemental file 1 — Supplemental material. Download spectrum.01966-22-s0001.pdf, PDF file, 0.8 MB [file spectrum.01966-22-s0001.pdf]

SUPPLEMENTAL MATERIAL

Supplemental material file 1: Table S1–S7

TABLE S1 Quantitative qPCR linearity tested via standard curves for each microorganism

Microorganism	Quantification range (Copy number, copies/μL)	Slope	Correlation coefficient (R^2)
Human cytomegalovirus	10^7 – 10^1	-3.324	0.999
Herpes simplex virus type 1	10^7 – 10^1	-3.510	0.998
Herpes simplex virus type 2	10^7 – 10^1	-3.321	0.998
Varicella-zoster virus	10^7 – 10^1	-3.613	0.994
Human herpesvirus 8	10^7 – 10^1	-3.161	0.998
Trichomonas vaginalis	10^7 – 10^1	-3.605	0.994
Ureaplasma parvum	10^7 – 10^1	-3.514	0.997
Ureaplasma urealyticum	10^7 – 10^1	-3.529	0.995
Haemophilus ducreyi	10^7 – 10^1	-3.621	0.993
Neisseria gonorrhoeae	10^7 – 10^1	-3.353	0.999
Treponema pallidum	10^7 – 10^1	-3.464	0.998
Chlamydia trachomatis	10^7 – 10^1	-3.486	0.997
Mycoplasma genitalium	10^7 – 10^1	-3.379	0.999
Klebsiella granulomatis	10^7 – 10^1	-3.357	0.999
Mycoplasma hominis	10^7 – 10^1	-3.203	0.997
Lactobacillus spp.	10^7 – 10^1	-3.588	0.999
Human RNase P (Internal control)	10^7 – 10^1	-3.430	0.995

TABLE S2 Diagnostic performance of the 16-plex MeltArray assay compared with the singleplex qPCR assays

MeltArray assay	Singleplex qPCR		Sensitivity	Specificity	PPV	NPV	Kappa value
	Positive	Negative	[% (95% CI)]	[% (95% CI)]	[% (95% CI)]	[% (95% CI)]	(95% CI)
Ureaplasma parvum							
Positive	396	6	95.19	98.86	98.51	96.31	0.9439
Negative	20	522	(92.67–97.04)	(97.54–99.58)	(96.75–99.32)	(94.45–97.56)	(0.9227–0.9652)
Human cytomegalovirus							
Positive	214	14	98.62	98.07	93.86	99.58	0.9500
Negative	3	713	(96.01–99.71)	(96.79–98.94)	(90.10–96.25)	(98.72–99.86)	(0.9265–0.9736)
Chlamydia trachomatis							
Positive	130	15	96.30	98.15	89.66	99.37	0.9162
Negative	5	794	(91.57–98.79)	(96.96–98.96)	(83.98–93.47)	(98.53–99.73)	(0.8799–0.9524)
Mycoplasma hominis							
Positive	106	14	98.15	98.33	88.33	99.76	0.9202
Negative	2	822	(93.47–99.78)	(97.21–99.08)	(81.82–92.72)	(99.05–99.94)	(0.8816–0.9589)
Human herpesvirus 8							
Positive	108	9	98.18	98.92	92.31	99.76	0.9449
Negative	2	825	(93.59–99.78)	(97.96–99.51)	(86.23–95.83)	(99.05–99.94)	(0.9126–0.9773)
Klebsiella granulomatis							
Positive	98	4	98.99	99.53	96.08	99.88	0.9722
Negative	1	841	(94.50–99.97)	(98.79–99.87)	(90.21–98.49)	(99.17–99.98)	(0.9478–0.9965)
Ureaplasma urealyticum							
Positive	73	0	97.33	100	100	99.77	0.9853
Negative	2	869	(90.70–99.68)	(99.58–100)	(100–100)	(99.11–99.94)	(0.9650–1.0000)
Treponema pallidum							
Positive	30	2	93.75	99.78	93.75	99.78	0.9353
Negative	2	910	(79.19–99.23)	(99.21–99.97)	(78.93–98.36)	(99.17–99.94)	(0.8722–0.9985)
Varicella-zoster virus							
Positive	23	0	92.00	100	100	99.78	0.9573
Negative	2	919	(73.97–99.02)	(99.60–100)	(100–100)	(99.18–99.94)	(0.8981–1.0000)
Mycoplasma genitalium							
Positive	18	0	90.00	100	100	99.78	0.9463
Negative	2	924	(68.30–98.77)	(99.60–100)	(100–100)	(99.20–99.94)	(0.8720–1.0000)

Trichomonas vaginalis							
Positive	16	1	100	99.89	94.12	100	0.9692
Negative	0	927	(79.41–100)	(99.40–100)	(69.29–99.13)	(100–100)	(0.9088–1.0000)
Neisseria gonorrhoeae							
Positive	17	0	94.44	100	100	99.89	0.9709
Negative	1	926	(72.71–99.86)	(99.60–100)	(100–100)	(99.28–99.98)	(0.9139–1.0000)
Herpes simplex virus type 1							
Positive	15	0	93.75	100	100	99.89	0.9672
Negative	1	928	(69.77–99.84)	(99.60–100)	(100–100)	(99.29–99.98)	(0.9030–1.0000)
Haemophilus ducreyi							
Positive	5	0	100	100	100	100	1.0000
Negative	0	939	(47.82–100)	(99.61–100)	(100–100)	(100–100)	(1.0000–1.0000)
Herpes simplex virus type 2							
Positive	3	0	75.00	100	100	99.89	0.8566
Negative	1	940	(19.41–99.37)	(99.61–100)	(100–100)	(99.42–99.98)	(0.5787–1.0000)

Abbreviations: PPV, positive predictive value; NPV, negative predictive value; CI, confidence interval.

TABLE S3 Association between microorganism quantity (normalized abundance ratio of microbial load with human RNase P load) and cervicitis versus healthy; vaginitis versus healthy

**Microorganism**	**Healthy (n=578) [n (%)][a]**	**Cervicitis (n=159) [n (%)][a]**		**Vaginitis (n=207) [n (%)][a]**	
			**p value[b]**		**p value[b]**
Lactobacillus spp.					
Detected, n (%)	576 (99.7)	149 (93.7)	**0.0001**	199 (96.1)	**0.002**
qPCR[c], median (IQR)	0.83 (0.61–0.90)	0.76 (0.62–0.84)	**<0.0001**	0.75 (0.57–0.84)	**<0.0001**
Trichomonas vaginalis					
Detected, n (%)	6 (1.0)	6 (3.8)	**0.024**	4 (1.9)	0.333
qPCR[c], median (IQR)	0.40 (0.37–0.57)	0.49 (0.40–0.71)	0.394	0.53 (0.51–0.59)	0.352
Mycoplasma genitalium					
Detected, n (%)	12 (2.1)	4 (2.5)	0.737	4 (1.9)	0.900
qPCR[c], median (IQR)	0.38 (0.36–0.46)	0.62 (0.51–0.76)	**0.030**	0.61 (0.56–0.77)	**0.008**
Ureaplasma urealyticum					
Detected, n (%)	41 (7.1)	13 (8.2)	0.643	21 (10.1)	0.165
qPCR[c], median (IQR)	0.52 (0.41–0.59)	0.60 (0.56–0.70)	**0.014**	0.62 (0.55–0.69)	**0.003**
Treponema pallidum					
Detected, n (%)	3 (0.5)	12 (7.5)	**<0.0001**	17 (8.2)	**<0.0001**
qPCR[c], median (IQR)	0.45 (0.43–0.57)	0.54 (0.46–0.63)	0.387	0.51 (0.49–0.54)	0.634
Neisseria gonorrhoeae					
Detected, n (%)	2 (0.3)	9 (5.7)	**0.0003**	7 (3.4)	**0.004**
qPCR[c], median (IQR)	0.37 (0.34–0.40)	0.39 (0.35–0.44)	0.637	0.45 (0.40–0.50)	0.143
Chlamydia trachomatis					
Detected, n (%)	42 (7.3)	32 (20.1)	**<0.0001**	61 (29.5)	**<0.0001**
qPCR[c], median (IQR)	0.26 (0.22–0.38)	0.25 (0.22–0.49)	0.853	0.26 (0.23–0.33)	0.778
Klebsiella granulomatis					
Detected, n (%)	47 (8.1)	22 (13.8)	**0.031**	30 (14.5)	**0.009**
qPCR[c], median (IQR)	0.36 (0.31–0.43)	0.34 (0.26–0.46)	0.607	0.36 (0.30–0.47)	0.909
Mycoplasma hominis					
Detected, n (%)	50 (8.7)	18 (11.3)	0.304	40 (19.3)	**0.0001**
qPCR[c], median (IQR)	0.23 (0.17–0.33)	0.42 (0.19–0.47)	**0.014**	0.36 (0.22–0.50)	**0.004**
Ureaplasma parvum					
Detected, n (%)	235 (40.7)	77 (48.4)	0.078	104 (50.2)	**0.017**
qPCR[c], median (IQR)	0.42 (0.35–0.50)	0.46 (0.38–0.52)	0.058	0.42 (0.34–0.50)	0.724
Haemophilus ducreyi					
Detected, n (%)	1 (0.2)	2 (1.3)	0.104	2 (1.0)	0.159
qPCR[c], median (IQR)	0.41 (0.41–0.41)	0.49 (0.45–0.52)	0.121	0.49 (0.44–0.53)	0.121

Human cytomegalovirus					
Detected, n (%)	123 (21.3)	34 (21.4)	0.978	60 (29.0)	**0.025**
qPCR[c], median (IQR)	0.42 (0.36–0.47)	0.44 (0.40–0.48)	0.133	0.45 (0.41–0.49)	**0.010**
Human herpesvirus 8					
Detected, n (%)	60 (10.4)	16 (10.1)	0.907	34 (16.4)	**0.023**
qPCR[c], median (IQR)	0.44 (0.41–0.49)	0.48 (0.43–0.51)	0.083	0.45 (0.41–0.50)	0.314
Varicella-zoster virus					
Detected, n (%)	11 (1.9)	6 (3.8)	0.172	8 (3.9)	0.123
qPCR[c], median (IQR)	0.32 (0.31–0.35)	0.43 (0.43–0.46)	**0.0009**	0.46 (0.41–0.50)	**0.0003**
Herpes simplex virus 1					
Detected, n (%)	12 (2.1)	2 (1.3)	0.508	2 (1.0)	0.312
qPCR[c], median (IQR)	0.34 (0.32–0.42)	0.40 (0.35–0.44)	0.440	0.30 (0.21–0.39)	0.440
Herpes simplex virus 2					
Detected, n (%)	3 (0.5)	0 (0)	0.662	1 (0.5)	0.950
qPCR[c], median (IQR)	0.36 (0.32–0.52)	0 (0)	0.076	0.34 (0.34–0.34)	0.248

Abbreviation: IQR, interquartile range.

[a]Data are number (percentage) of subjects.

[b]Chi-square test and Fisher's exact test (binary variables), Mann-Whitney *U* test (continuous variables). Bold indicates statistical significance at $p < 0.05$.

[c]Normalized abundance ratio (NAR): Microbial load ($\log_{10}$ copies/mL) / Human RNase P load ($\log_{10}$ copies/mL).

TABLE S4 Multivariable logistic analysis showing the association between microorganisms by qualitative and quantitative qPCR and symptomatic women with cervicitis and vaginitis

Variables	MeltArray assay and normalized qPCR (NAR above the cut-off)							
	Cervicitis				Vaginitis			
	full model without selection		stepwise selection[a]		full model without selection		stepwise selection[a]	
	Adjusted OR[b] (95% CI)	p value[c]	Adjusted OR[f] (95% CI)	p value[c]	Adjusted OR[b] (95% CI)	p value[c]	Adjusted OR[g] (95% CI)	p value[c]
**Age, years**								
16–25	Reference	-	Reference	-	Reference	-	Reference	-
26–35	0.19 (0.08–0.44)	**<0.0001**	0.19 (0.08–0.45)	**<0.0001**	0.33 (0.14–0.78)	**0.012**	0.28 (0.12–0.63)	**0.002**
36–45	0.23 (0.10–0.56)	**0.001**	0.23 (0.10–0.55)	**0.001**	0.32 (0.13–0.76)	**0.010**	0.29 (0.12–0.68)	**0.005**
46–55	0.15 (0.06–0.41)	**<0.0001**	0.15 (0.06–0.39)	**<0.0001**	0.21 (0.08–0.55)	**0.001**	0.19 (0.08–0.49)	**0.001**
≥ 56	0.06 (0.01–0.34)	**0.002**	0.07 (0.01–0.34)	**0.001**	0.14 (0.03–0.57)	**0.006**	0.12 (0.03–0.50)	**0.003**
**Microorganism, MeltArray assay[d]**								
Ureaplasma parvum	1.05 (0.61–1.79)	0.864	-	-	0.82 (0.37–1.83)	0.629	-	-
Chlamydia trachomatis	2.12 (1.14–3.97)	**0.018**	2.36 (1.34–4.18)	**0.003**	2.67 (1.19–5.98)	**0.017**	4.56 (2.82–7.38)	**<0.0001**
Mycoplasma hominis	0.49 (0.21–1.16)	0.104	-	-	1.13 (0.60–2.16)	0.704	-	-
Klebsiella granulomatis	2.03 (1.02–4.05)	**0.045**	2.23 (1.21–4.11)	**0.010**	1.78 (0.95–3.34)	0.074	2.17 (1.25–3.78)	**0.006**
Ureaplasma urealyticum	0.52 (0.13–2.00)	0.339	-	-	0.89 (0.32–2.48)	0.823	-	-
Treponema pallidum	10.73 (2.53–45.52)	**0.001**	10.99 (2.70–44.70)	**0.001**	2.43 (0.36–16.38)	0.362	-	-
Neisseria gonorrhoeae	9.30 (1.49–58.01)	**0.017**	20.82 (4.02–107.95)	**<0.0001**	3.91 (0.50–30.42)	0.193	7.32 (1.33–40.43)	**0.022**
Human cytomegalovirus	0.57 (0.25–1.30)	0.180	-	-	0.52 (0.21–1.30)	0.161	-	-
Human papillomavirus	2.59 (1.51–4.43)	**0.001**	2.54 (1.51–4.28)	**<0.0001**	1.33 (0.75–2.35)	0.335	-	-
Human herpesvirus 8	0.53 (0.20–1.38)	0.194	-	-	1.04 (0.46–2.38)	0.920	-	-
Trichomonas vaginalis	0.52 (0.04–6.71)	0.615	-	-	-	-	-	-
**Microorganism, normalized qPCR (NAR above the cut-off)[e]**								
Lactobacillus spp.	0.37 (0.22–0.61)	**<0.0001**	0.40 (0.25–0.65)	**<0.0001**	0.49 (0.32–0.74)	**0.001**	0.50 (0.33–0.73)	**<0.0001**
Trichomonas vaginalis	5.21 (0.17–159.51)	0.345	-	-	-	-	5.54 (0.92–33.48)	0.062
Mycoplasma genitalium	-	-	5.52 (0.97–31.53)	0.055	-	-	-	-
Ureaplasma urealyticum	4.45 (0.78–25.41)	0.093	-	-	2.63 (0.65–10.69)	0.176	2.64 (1.02–6.78)	**0.045**
Treponema pallidum	-	-	-	-	17.75 (1.07–294.89)	**0.045**	51.64 (6.36–419.39)	**<0.0001**
Chlamydia trachomatis	4.44 (0.61–32.40)	0.142	-	-	2.01 (0.78–5.17)	0.147	-	-
Klebsiella granulomatis	1.08 (0.24–4.96)	0.918	-	-	2.50 (0.65–9.64)	0.182	-	-
Mycoplasma hominis	9.13 (1.99–41.92)	**0.004**	4.94 (1.44–16.89)	**0.011**	8.01 (2.21–29.04)	**0.002**	9.02 (2.85–28.53)	**<0.0001**
Ureaplasma parvum	2.38 (1.24–4.56)	**0.009**	2.26 (1.33–3.84)	**0.002**	1.48 (0.66–3.32)	0.343	-	-
Human cytomegalovirus	1.66 (0.64–4.34)	0.301	-	-	3.04 (1.16–7.99)	**0.024**	1.72 (1.09–2.73)	**0.021**
Human herpesvirus 8	2.81 (0.80–9.89)	0.109	-	-	2.41 (0.89–6.55)	0.085	2.57 (1.35–4.88)	**0.004**

Abbreviations: aOR, adjusted odds ratio; CI, confidence interval

[a]The p-value threshold of 0.05 is required to allow a factor into the model ($p < 0.05$), and a significance level of 0.10 ($p < 0.10$) is required for a factor to stay in the logistic model.

[b]Adjustment was made in a multivariable model stratified by cohort for age (five categories).

[c]Bold indicate the adjusted OR with significance at $p < 0.05$.

[d]Qualitative PCR test using 16-plex MeltArray assay.

[e]Cut-off values were set according to the Youden index calculated using MedCalc. The microorganism detection rate was determined based on NAR above the cut-off.

[f]Prediction model for cervicitis, natural log of odds (O) = -0.443 (intercept) + (age 26–35 × -1.369) + (age 36–45 × -1.087) + (age 46–55 × -1.542) + (age ≥56 × -2.472) + (HPV × 0.813) + (NG × 2.960) + (TP × 2.769) + (CT × 0.812) + (KG × 0.841) + (LAC_NAR × -0.933) + (MH_NAR × 1.653) + (UP_NAR × 0.669) + (MG_NAR × 1.729)

[g]Prediction model for vaginitis, natural log of odds (O) = -0.394 (intercept) + (age 26–35 × -1.050) + (age 36–45 × -1.043) + (age 46–55 × -1.441) + (age ≥56 × -1.568) + (NG × 1.959) + (CT × 1.492) + (KG × 0.750) + (LAC_ NAR × -0.673) + (UU_NAR × 0.948) + (TP_NAR × 3.868) + (MH_NAR × 2.249) + (HCMV_NAR × 0.488) + (HHV-8_NAR × 0.947).

TABLE S5 Comparison of AUC for the combine qualitative and quantitative qPCR for diagnosing both cervicitis and vaginitis

	**Cervicitis**		**Vaginitis**	
	**AUC (95% CI)**	**p value[d]**	**AUC (95% CI)**	**p value[d]**
Qualitative, MeltArray assay[a]	0.74 (0.71–0.78)	**0.0001**	0.75 (0.72–0.78)	0.135
Quantitative, normalized qPCR (NAR above the cut-off)[b]	0.76 (0.73–0.79)	**0.024**	0.76 (0.73–0.79)	0.161
Combined test[c]	0.80 (0.77–0.83)	-	0.78 (0.75–0.81)	-

Abbreviations: AUC, area under the receiver operating characteristic curve; CI, confidence interval

[a]Qualitative test using 16-plex MeltArray assay.

[b]Cut-off values were set according to the Youden index calculated using MedCalc, and the detection rate of NAR above the cut-off was calculated for the analysis.

[c]Simultaneous qualitative and quantitative qPCR screening to discriminate between cases and controls.

[d]Pairwise comparison of ROC curves between qualitative MeltArray assay and combined test, and between quantitative qPCR and combined test. Bold indicates significance at $p < 0.05$.

TABLE S6 Melting temperature of 16-plex MeltArray assay

Channel	Microorganism	T_m (°C)
**FAM**	Human cytomegalovirus	61.4
	Herpes simplex virus type 1	65.5
	Herpes simplex virus type 2	69.1
	Trichomonas vaginalis	74.5
	Ureaplasma urealyticum	80.4
**ROX**	*Haemophilus ducreyi*	56.9
	Neisseria gonorrhoeae	67.1
	Treponema pallidum	73.3
	Chlamydia trachomatis	78.9
	Mycoplasma genitalium	85.3
**Cy5**	*Ureaplasma parvum*	60.2
	Klebsiella granulomatis	66.7
	Varicella-Zoster virus	70.9
	Mycoplasma hominis	79.2
	Human herpesvirus 8	84.8
**HEX**	Human RNase P (Internal control)	64.9

TABLE S7 Sequence of primers and probes for singleplex qPCR for quantification of each microorganism

Microorganism	Target	Nucleotide sequence (5'→3')	Ref.
Human cytomegalovirus	*IE72*	F: GCTCCTCTGATTCTCTGGTGTC<br>R: ACTGTTCTCAGCCACAATTACT<br>P: FAM-CCCCAGAGTCCCCTGTACCCGC-BHQ1	[1]
Herpes simplex virus type 1	*gB*	F: CGCATCAAGACCACCTCCTC<br>R: GCTCGCACCACGCGA<br>P: ROX-TGGCAACGCGGCCCAAC-BHQ2	[2]
Herpes simplex virus type 2	*gB*	F: CGCATCAAGACCACCTCCTC<br>R: GCTCGCACCACGCGA<br>P: FAM-CGGCGATGCGCCCCAG-BHQ1	[2]
Varicella-zoster virus	*ORF29*	F: AACTTTTACATCCAGCCTGGCG<br>R: GAAAACCCAAACCGTTCTCGAG<br>P: FAM-TGTCTTTCACGGAGGCAAACACGT-BHQ1	[2]
Human herpesvirus 8	*ORF65*	F: CCTCTGGTCCCCATTCATTG<br>R: CGTTTCCGTCGTGGATGAG<br>P: ROX-CCGGCGTCAGACATTCTCACAACC-BHQ2	[2]
Trichomonas vaginalis	*repeated DNA*	F: AAGATGGGTGTTTTAAGCTAGATAAGGT<br>R: CGTCTTCAAGTATGCCCCAGTAC<br>P: FAM-CCGAAGTTCATGTCCTCTCCAAGCGT-BHQ1	[3]
Ureaplasma parvum	*ureA*	F: AATTCATGCTTATCATACAGAAGG<br>R: ATGGGCATATTTAACAGTTTCTAG<br>P: FAM-AGCATGTCCTCCACCAG-BHQ1	[4]
Ureaplasma urealyticum	*ureB*	F: CTGGTGATAGCGTTAGATTAGG<br>R: GTTCATCCCCATACCTTCAC<br>P: ROX-ACCACCACCGAAGACAG-BHQ2	[4]
Haemophilus ducreyi	*16S rRNA*	F: ACATCCATAGAAGAACTCAGAGATGA<br>R: TTGAGTTCCCATCAYTACATGCT<br>P: FAM-GTGCCTTCGGGAACTATGTGACAGGT-BHQ1	[5]
Neisseria gonorrhoeae	*porA*	F: CCGGAACTGGTTTCATCTGATTAC<br>R: GGTTTCAGCGGCAGCATTCAAT<br>P: FAM-AGTAGCAGGCGTATAGGCGGACTT-BHQ1	[6]
Treponema pallidum	*polA*	F: GGTAGAAGGGAGGGCTAGTA<br>R: CTAAGATCTCTATTTTCTATAGGTATGG<br>P: FAM-GGAGTTGAAGACGAGTGCTGTGT-BHQ1	[7]
Chlamydia trachomatis	*omcB*	F: GACACCAAAGCGAAAGACAACAC<br>R: ACTCATGAACCGGAGCAACCT<br>P: FAM-AAGCAAAAAAGCAAGAAAAAACCACAGCAAAGAG-BHQ1	[8]
Mycoplasma genitalium	*pdhD*	F: CGGATCAAGACCAAGATACTTAACTTT<br>R: AGCTTGGGTTGAGTCAATGATAAAC<br>P: FAM-CCAGGGTTTGAAAAAGCACAACAAGCTG-BHQ1	[9]
Klebsiella granulomatis	*phoE*	F: TCCTCTGCCCAGACCGATAACTTTATG<br>R: CCAGGTAGATATTGTTGGCGTCA<br>P: FAM-GCCGTCAGCGCAGCCTACACCAGC-BHQ1	[10]
Mycoplasma hominis	*yidC*	F: TCACTAAACCGGGTATTTTCTAACAA<br>R: TTGGCATATATTGCGATAGTGCTT<br>P: FAM-CTACCAATAATTTTAATATCTGTCGG-BHQ1	[11]
Lactobacillus spp.	*rplk*	F: GCACCTCCAGTTGGTCCTG<br>R: CATGCTTCTAGCGGTACCTTCA<br>P: FAM-TTCATTACTAAGACTCCACC-BHQ1	[12]
Human RNase P (Internal control)	*RPP30*	F: GGCGGTGTTTGCAGATTTG<br>R: GAGCGGCTGTCTCCACAAGT<br>P: FAM-TCTGACCTGAAGGCTCTGCGCG-BHQ1	[13]

References

1. Xiang Z, Fang F, Zheng H, Xu Y, Liu J, Nie X. 2005. Comparison between real-time PCR technique and rapid culture assay for the quantitative detection of human cytomegalovirus in urine. *J Clin Pediatrics* 07.
2. Sugita S, Shimizu N, Watanabe K, Mizukami M, Morio T, Sugamoto Y, Mochizuki M. 2008. Use of multiplex PCR and real-time PCR to detect human herpes virus genome in ocular fluids of patients with uveitis. *Br J Ophthalmol* 92:928–932.
3. Schirm J, Bos PA, Roozeboom-Roelfsema IK, Luijt DS, Moller LV. 2007. *Trichomonas vaginalis* detection using real-time TaqMan PCR. *J Microbiol Methods* 68:243–247.
4. Tang J, Zhou L, Liu X, Zhang C, Zhao Y, Wang Y. 2011. Novel multiplex real-time PCR system using the SNP technology for the simultaneous diagnosis of *Chlamydia trachomatis*, *Ureaplasma parvum* and *Ureaplasma urealyticum* and genetic typing of serovars of *C. trachomatis* and *U. parvum* in NGU. *Mol Cell Probe* 25:55–59.
5. Glatz M, Juricevic N, Altwegg M, Bruisten S, Komericki P, Lautenschlager S, Weber R, Bosshard PP. 2014. A multicenter prospective trial to asses a new real-time polymerase chain reaction for detection of *Treponema pallidum*, herpes simplex-1/2 and *Haemophilus ducreyi* in genital, anal and oropharyngeal ulcers. *Clin Microbiol Infect* 20: O1020-7.
6. Hjelmevoll SO, Olsen ME, Sollid JU, Haaheim H, Unemo M, Skogen V. 2006. A fast real-time polymerase chain reaction method for sensitive and specific detection of the *Neisseria gonorrhoeae* porA pseudogene. *J Mol Diagn* 8:574–581.
7. Koek AG, Bruisten SM, Dierdorp M, Dam APV, Templeton K. 2006. Specific and sensitive diagnosis of syphilis using a real-time PCR for *Treponema pallidum*. *Clin Microbiol Infect* 12:1233–1236.
8. Pickett MA, Everson JS, Pead PJ, Clarke IN. 2005. The plasmids of *Chlamydia trachomatis* and *Chlamydophila pneumoniae* (N16): accurate determination of copy number and the paradoxical effect of plasmid-curing agents. *Microbiol* 151:893–903.
9. Müller EE, Venter JME, Magooa MP, Morrison C, Lewis DA, Mavedzenge SN. 2012. Development of a rotor-gene real-time PCR assay for the detection and quantification of *Mycoplasma genitalium*. *J Microbiol Methods* 88:311–315.
10. Mackay IM, Harnett G, Jeoffreys N, Bastian I, Sriprakash KS, Siebert D, Sloots TP. 2006. Detection and discrimination of herpes simplex viruses, *Haemophilus ducreyi*, *Treponema pallidum*, and *Calymmatobacterium (Klebsiella) granulomatis* from genital ulcers. *Clin Infect Dis* 42:1431–1438.
11. Férandon C, Peuchant O, Janis C, Benard A, Renaudin H, Pereyre S, Bébéar C. 2011. Development of a real-time PCR targeting the *yidC* gene for the detection of *Mycoplasma hominis* and comparison with quantitative culture. *Clin Microbiol Infect* 17:155–159.
12. Demkin VV, Koshechkin SI, Slesarev A. 2017. A novel real-time PCR assay for highly specific detection and quantification of vaginal lactobacilli. *Mol Cell Probe* 32:33–39.
13. Barik MR, Rath S, Modi R, Rana R, Reddy MM, Basu S. 2018. Normalised quantitative polymerase chain reaction for diagnosis of tuberculosis-associated uveitis. *Tuberculosis* 110:30–35.

Supplemental material file 2: FIG S1–S4

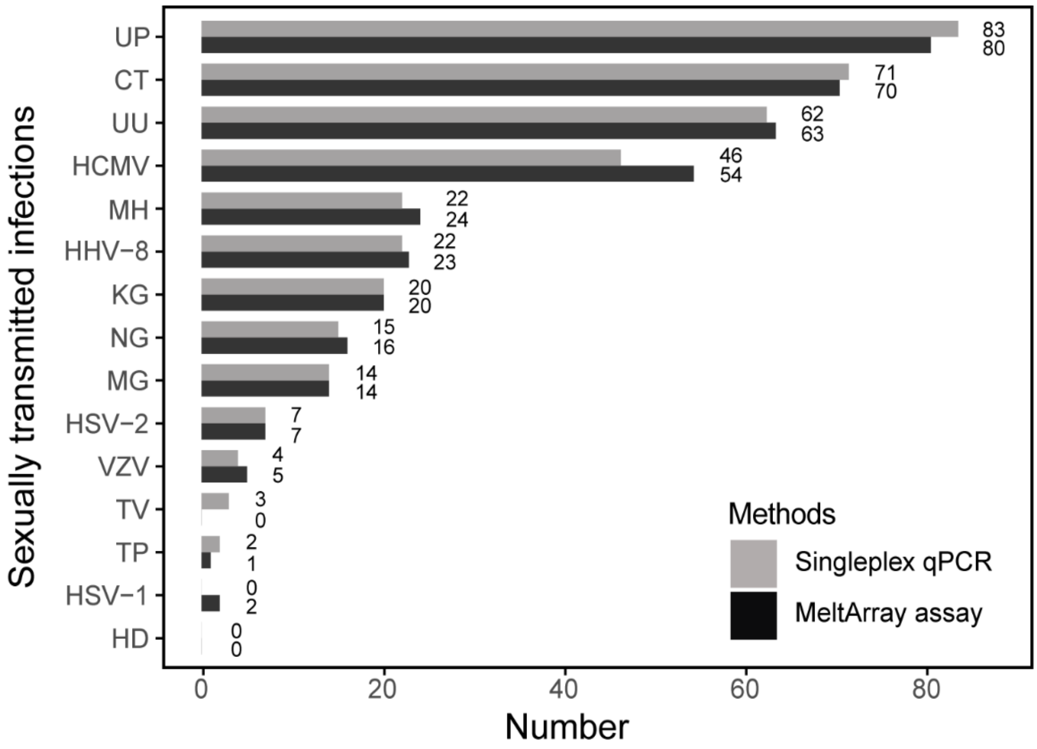

FIG S1. Diagnostic performance of the 16-plex MeltArray assay compared with the singleplex qPCR. Number of STI pathogens detected by both of the 16-plex MeltArray and singleplex qPCR assays for the clinical evaluation from 203 swab samples.

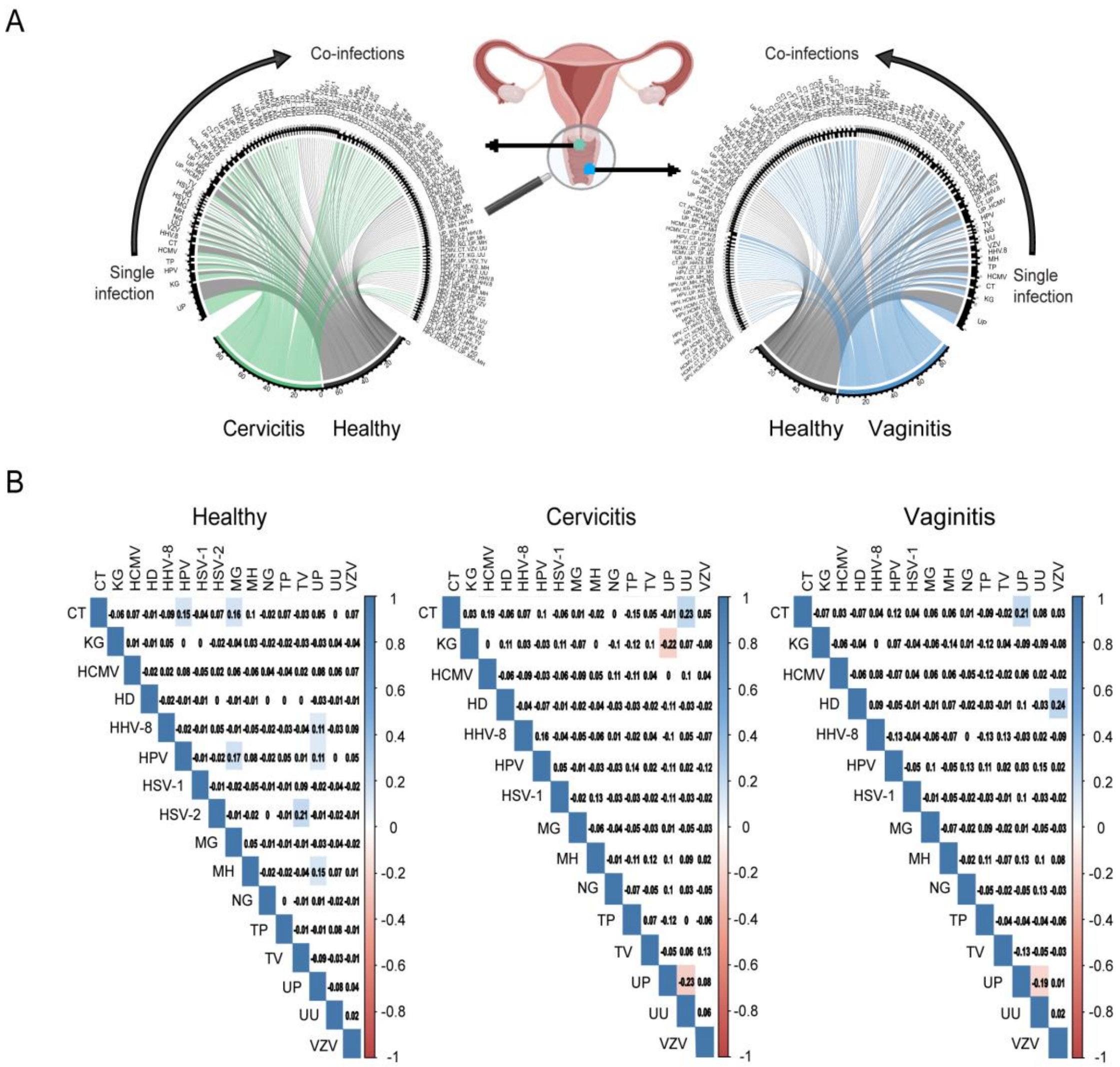

FIG S2. Co-infections pattern and Spearman's correlation coefficient for all detected STI pathogens in healthy, cervicitis, and vaginitis subjects. (A) Chord diagram represents the single and co-infections of STI pathogens among cervicitis versus healthy, and vaginitis versus healthy. **(B)** Potential correlations between the coexistence of STI pathogens explored using Spearman's correlation coefficients. Strong positive correlations are indicated by dark blue and strong negative correlations by dark red. Correlation is significant at $p < 0.01$ and shaded in blue and red, respectively.

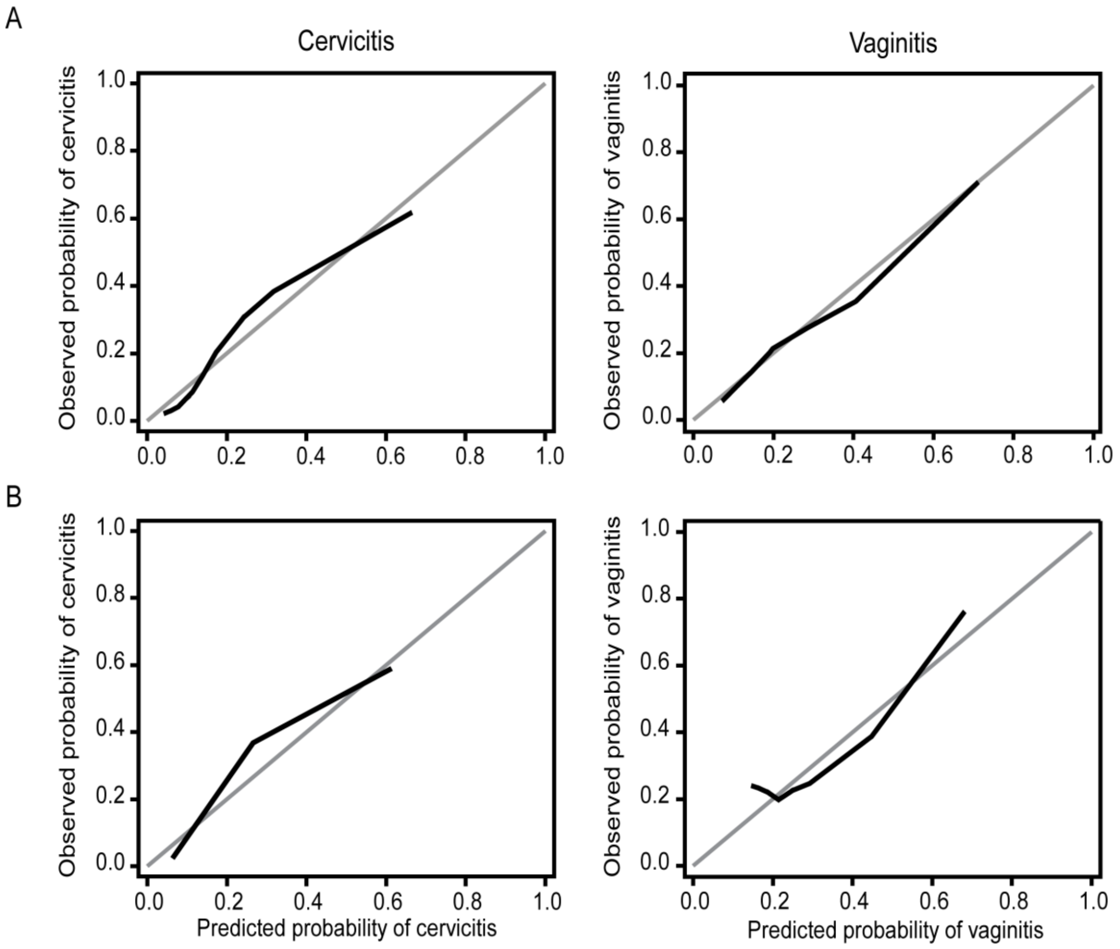

FIG S3. Calibration plots of the logistic regression prediction models. (A) Goodness-of-fit for simultaneous qualitative and quantitative screening of cervicitis and vaginitis from the derivation dataset. **(B)** Goodness-of-fit for simultaneous qualitative and quantitative screening of cervicitis and vaginitis from the validation dataset. Plots show how well the predicted probabilities (x-axis) agree with observed probabilities (y-axis). For perfect agreement, the black line falls on the ideal diagonal line (optimal calibration).

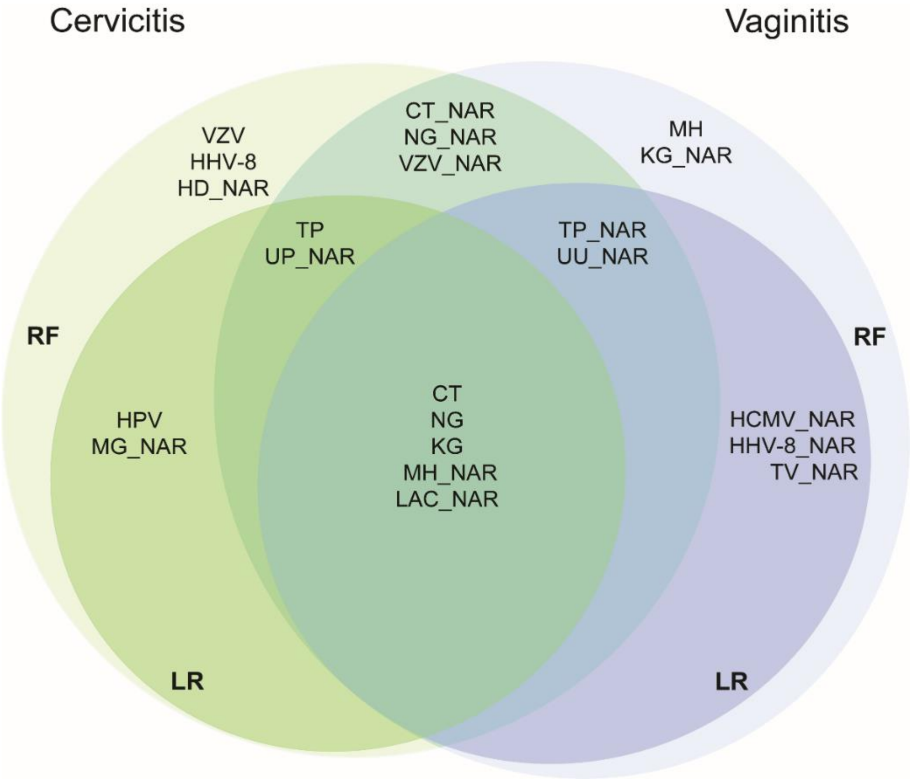

FIG S4. Venn diagram of the variables (features) present in cervicitis and vaginitis via logistic regression (LR) and random forest (RF) models using both qualitative and quantitative datasets.

Supplemental material file 3: R source code

Random forest code (Cervicitis)

```
>library(Boruta)
>library(mlbench)
>library(caret)
>library(randomForest)
>library(pROC)
```

Derivation datatset

```
>dataHCRF<-read.csv(choose.files(), header = T)
>str(dataHCRF)
>dataHCRF$COHORT <- as.factor(dataHCRF$COHORT)
>table(dataHCRF$COHORT)
>set.seed(111)
>boruta <- Boruta(COHORT ~ ., data = dataHCRF, doTrace = 2, maxRuns =500)
>print(boruta)
>plot(boruta, las = 2, cex.axis = 0.5)
>plotImpHistory(boruta)
>getNonRejectedFormula(boruta)
>getConfirmedFormula(boruta)
>bor <- TentativeRoughFix(boruta)
>print(bor)
>attStats(boruta)

>set.seed(222)
>ind <- sample(2, nrow(dataHCRF), replace = T, prob = c(0.7, 0.3))
>train <- dataHCRF[ind==1,]
>test <- dataHCRF[ind==2,]

>set.seed(333)
>rf34 <- randomForest(COHORT~., data = train)
>rf34
>plot(rf34)
>pTest <- predict(rf34, test)
>confusionMatrix(pTest, test$COHORT)
```

```
>t <- tuneRF(train[,-34], train[, 34],
            stepFactor = 0.5, plot = TRUE, ntreeTry = 300,
            trace = TRUE, improve = 0.05)

>rf34 <- randomForest(COHORT~., data = train,
                     ntree = 300,
                     mtry = 5,
                     importance = TRUE,
                     proximity = TRUE)
>p1 <- predict(rf34, test)
>confusionMatrix(p1, test$COHORT)

>rf18con <- randomForest(COHORT ~ AGE + UU_NAR + HD_NAR + NG_NAR + TP_NAR +
CT_NAR + MG_NAR + UP_NAR + VZV_NAR + MH_NAR + LAC_NAR + HPV + NG + TP +
CT + KG + VZV + HHV8,
                      data = train,
                      ntree = 600,
                      mtry = 5,
                      importance = TRUE,
                      proximity = TRUE)
>p <- predict(rf18con, test)
>confusionMatrix(p, test$COHORT)
>varImpPlot(rf18con)
>importance(rf18con)
>varUsed(rf18con)
```

Validation datatset

```
>dataHCRFvalid<-read.csv(choose.files(), header = T)
>set.seed(333)
>rfcon <- randomForest(COHORT ~ AGE + UU_NAR + HD_NAR + NG_NAR + TP_NAR +
CT_NAR + MG_NAR + UP_NAR + VZV_NAR + MH_NAR + LAC_NAR + HPV + NG + TP +
CT + KG + VZV + HHV8,
                      data = dataHCRFvalid,
                      ntree = 300,
                      mtry = 5,
                      importance = TRUE,
                      proximity = TRUE)
>p <- predict(rfcon, dataHCRFvalid, type="prob")
>p2 <- predict(rfcon, dataHCRFvalid)
```

```
>confusionMatrix(p2, dataHCRFvalid$COHORT, positive = "Patient")
>ran_roc<-roc(dataHCRFvalid$COHORT, levels= c("Healthy", "Patient"), direction='<', as.numeric(p2), ci=T,auc = T)
>plot(ran_roc, print.auc=TRUE)
```

Random forest code (Vaginitis)

```
>library(Boruta)
>library(mlbench)
>library(caret)
>library(randomForest)
>library(pROC)
```

Derivation datatset

```
>dataHVRF<-read.csv(choose.files(), header = T)
>str(dataHVRF)
>dataHVRF$COHORT <- as.factor(dataHVRF$COHORT)
>table(dataHVRF$COHORT)
>set.seed(111)
>boruta <- Boruta(COHORT ~ ., data = dataHVRF, doTrace = 2, maxRuns =500)
>print(boruta)
>plot(boruta, las = 2, cex.axis = 0.5)
>plotImpHistory(boruta)
>getNonRejectedFormula(boruta)
>getConfirmedFormula(boruta)
>borV <- TentativeRoughFix(boruta)
>print(borV)
>attStats(boruta)

>set.seed(222)
>ind <- sample(2, nrow(dataHVRF), replace = T, prob = c(0.7, 0.3))
>train <- dataHVRF[ind==1,]
>test <- dataHVRF[ind==2,]

>set.seed(333)
>rf34 <- randomForest(COHORT~., data = train)
>plot(rf34)
>pTest <- predict(rf34, test)
>confusionMatrix(pTest, test$COHORT)

>t <- tuneRF(train[,-34], train[, 34],
        stepFactor = 0.5, plot = TRUE, ntreeTry = 300,
        trace = TRUE, improve = 0.05)
```

```
>rf34 <- randomForest(COHORT~., data = train,
                      ntree = 300,
                      mtry = 5,
                      importance = TRUE,
                      proximity = TRUE)
>pTest <- predict(rf34, test)
>confusionMatrix(pTest, test$COHORT)

>rf18 <- randomForest(COHORT ~ Age + NG + TP + CT + KG + MH + HCMV_NAR + TV_NAR
           + UU_NAR + NG_NAR + TP_NAR + CT_NAR + UP_NAR + KG_NAR + VZV_NAR
           + MH_NAR + HHV.8_NAR + LAC_NAR,
                      data = train, ntree = 300,
                       mtry = 5,
                       importance = TRUE,
                       proximity = TRUE)
>p <- predict(rf18, test)
>confusionMatrix(p, test$COHORT)
>varImpPlot(rf18)
>importance(rf18)
>varUsed(rf18)
```

Validation dataset

```
>dataHVRFvalid<-read.csv(choose.files(), header = T)
>set.seed(333)
>rf18 <- randomForest(COHORT ~ Age + NG + TP + CT + KG + MH + HCMV_NAR + TV_NAR
            + UU_NAR + NG_NAR + TP_NAR + CT_NAR + UP_NAR + KG_NAR +
            VZV_NAR + MH_NAR + HHV.8_NAR + LAC_NAR,
                       data = dataHVRFvalid, ntree = 300,
                       mtry = 5,
                       importance = TRUE,
                       proximity = TRUE)
>p <- predict(rf18, dataHVRFvalid, type="prob")
>p2 <- predict(rf18, dataHVRFvalid)
>confusionMatrix(p2, dataHVRFvalid$COHORT , positive = "Patient")
>ran_roc<-roc(dataHVRFvlid$COHORT, levels= c("Healthy", "Patient"), direction='<',
as.numeric(p2), ci=T, auc= T)
>plot(ran_roc, print.auc=TRUE)
```
